# Contagious itch: what we know and what we would like to know

**DOI:** 10.3389/fnhum.2015.00057

**Published:** 2015-02-11

**Authors:** C. Schut, S. Grossman, U. Gieler, J. Kupfer, G. Yosipovitch

**Affiliations:** ^1^Department of Dermatology and Temple Itch Center, Temple University School of MedicinePhiladelphia, PA, USA; ^2^Institute of Medical Psychology, Justus-Liebig-UniversityGiessen, Germany; ^3^Department of Dermatology, University ClinicGiessen, Germany

**Keywords:** contagious itch, atopic dermatitis, itch, mirror neurons, psychological factors

## Abstract

All humans experience itch in the course of their life. Even a discussion on the topic of itch or seeing people scratch can evoke the desire to scratch. These events are coined “contagious itch” and are very common. We and others have shown that videos showing people scratching and pictures of affected skin or insects can induce itch in healthy persons and chronic itch patients. In our studies, patients with atopic dermatitis (AD) were more susceptible to visual itch cues than healthy. Also, personality traits like agreeableness and public self-consciousness were associated with induced scratching in skin patients, while neuroticism correlated with induced itch in healthy subjects. The underlying course of contagious itch is not yet fully understood. It is hypothesized that there are human mirror neurons that are active when we imitate actions and/or negative affect. Until now, there has been only limited data on the mechanisms of brain activation in contagious itch though. We have barely begun to understand the underlying physiological reactions and the triggering factors of this phenomenon. We summarize what we currently know about contagious itch and provide some suggestions what future research should focus on.

## Itch—a frequently underestimated phenomenon

Everybody experiences itch in his or her life. Itch is a bodily sensation that is described as unpleasant and accompanied by the desire to scratch (Hafenreffer, 1660). It is a symptom that is considered to be rather annoying and bothersome (Dawn et al., 2009), but the subsequent scratching resulting in itch relief has been associated with positive feelings like pleasure (Mochizuki et al., 2014b).

Itch is a frequent symptom in the general population and among those with disease (Weisshaar and Matterne, 2014). About 8–17% of the general population (Dalgard et al., 2004; Matterne et al., 2009, 2011, 2013; Ständer et al., 2010) and up to 100% of patients with skin diseases like atopic dermatitis (AD) chronic idiopathic urticaria and psoriasis suffer from itch (Hanifin and Rajka, 1980; Yosipovitch et al., 2002; Reich et al., 2010). Pruritus has been documented in 10–70% of patients with kidney disease and 15–100% of patients with liver disease (Weisshaar and Dalgard, 2009).

In studies investigating the underlying physiological causes and consequences of itch, researchers generally use pruritogens like histamine, serotonin or cowhage to induce itch in both animals and humans (e.g., LaMotte et al., 2009; van Laarhoven et al., 2011; Papoiu et al., 2013). The application of pruritogens leads to intense feelings of itch and scratching behavior (Yamaguchi et al., 1999; Davidson et al., 2007). There is also a non-skin-manipulating method to induce itch by the presentation of certain sounds, pictures or videos. This method is referred to as audiovisual transmission of itch and is the basis of contagious itch.

## Audiovisual transmission of itch in humans and nonhuman primates

It can be easily acknowledged that people feel itch when they see another individual exhibiting scratching behavior or when they are discussing itch. However, this phenomena had not been studied until the last decade. The first study demonstrating that itch can be induced by visual stimuli was published by Niemeier et al. (2000). In this study, a human audience observed two slide-supported presentations. The first presentation was an itch-inducing lecture (including e.g., pictures of insects, scratch marks, allergic reactions) entitled, “itching—what is behind it?”, while the second presentation focused on relaxation (including e.g., pictures on baby skin or children with their mothers). Analysis of the number of scratch movements during the presentations revealed a significant increase in scratching during the “itch lecture” compared to the “relaxation lecture”. Moreover, questionnaire data revealed that increased itch was reported following the “itch lecture”.

A decade later, a similar approach was used to analyze whether visual stimuli without auditory accompaniment would also be able to induce itch (Ogden and Zoukas, 2009). In this study, groups of students were shown video clips to induce coldness, pain or itch. The itch-inducing video contained pictures of head lice moving across hair strands and of people scratching their heads. During the “itch video”, the students not only scratched more often, but also reported higher levels of itchiness than during the videos on coldness and pain (Ogden and Zoukas, 2009). Unfortunately, this study lacked a control condition. It would have improved the study if scratch movements and itch intensity were recorded during a baseline situation, when subjects were presented with a “neutral video”.

**Figure 1:**
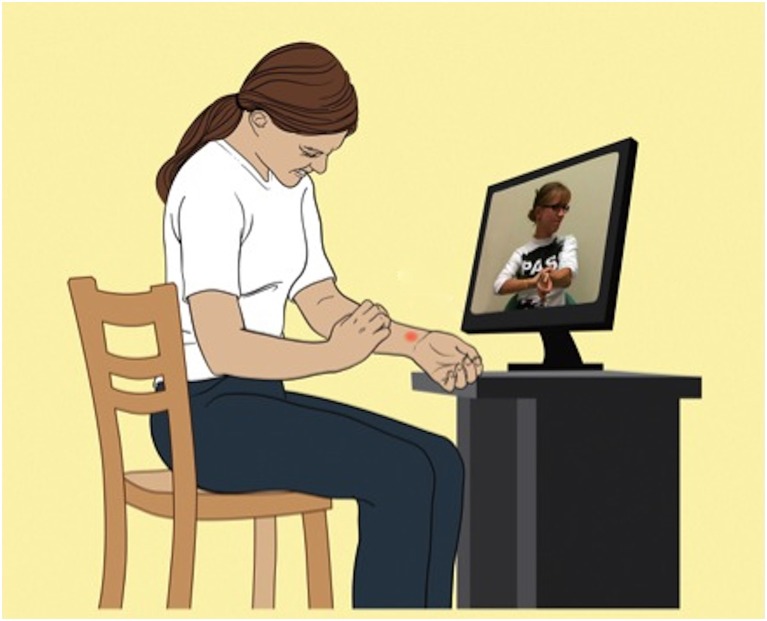
**Seeing another person scratching leads to a significant increase in scratching**. This phenomenon is more pronounced in patients with chronic itch (patients with atopic dermatitis) compared to healthy controls (Modified picture from Papoiu et al., 2011).

More recently, studies that included a control video provided further support that itch and scratching can be induced by visual itch stimuli. One investigation used videos showing other people scratching in order to induce itch in healthy humans and AD-patients (Papoiu et al., 2011). Both groups reported increased itch while watching short video clips of other people scratching (also see Figure 1) compared to watching a control video where the same people were sitting idle. However, the increase in itch intensity and in the number of scratch movements was significantly higher in AD-patients (Papoiu et al., 2011). Schut et al. (2014) were able to replicate the finding that patients suffering from AD are more prone to itch-inducing (audio-)visual stimuli than healthy controls.

It has also been shown that the body parts that are itchy due to visual stimuli differ between chronic itch patients and healthy controls. Papoiu et al. (2011) demonstrated that AD-patients scratched at body sites distal from the body part that was being scratched in the video, while healthy controls scratched body parts that were proximal to the body part that was being scratched in the video. Ward et al. (2013) supported this conclusion by maintaining that healthy participants preferentially scratch their heads (a proximal location), even when visual stimuli depict scratching of the chest and arms. Feneran et al. (2013) also demonstrated a similar pattern of macaque monkeys scratching parts of the body that were not identical to those attended to in neighboring scratching cagemates, as well as in videos of other monkeys scratching. This data suggests that contagious itch is not location-specific and a behavioral response that occurs in non-human primates and other mammals.

Rather than comparing the scratch and itch response between skin patients and healthy controls, Lloyd et al. (2013) were interested in the scratch- and itch response to *different* itch-related static images (e.g., insects or skin conditions) in healthy participants. The itch-related stimuli were delineated into the categories “skin contact” (e.g., ants crawling on a hand), “skin response” (e.g., scratching an insect bite) or “context only” (e.g., merely looking at insects). This study showed that the scratch responses increased the most, when subjects saw pictures of the “skin response” category, while the itch response was most intense to pictures showing itch stimuli of the category “context only” or “skin contact”. The subjects were also asked to report how itchy they themselves felt, as well as how itchy they thought the subject in the static image felt. Participants reported high itch sensations for both themselves as well as what they imagined the subjects in the pictures would feel, which may illustrate the role of empathy in contagious itch.

One of the candidates for empathic processing is the mirror neuron system (Iacoboni, 2009). The origin of this system is not fully understood yet: On the one hand it is argued that mirror neurons are inherent and serve to understand the actions of others, while on the other hand it is hypothesized that mirror neurons develop as a consequence of associative learning, which many times occurs in social interactions (Rizzolatti and Craighero, 2004; Hickok, 2009; Heyes, 2010). Mirror neurons were originally observed in the Ventral Premotor Area (VPA) of monkeys and are a specific type of motor cell that fires not only when the animal makes a specific movement, but also when it observes the same movement being carried out (di Pellegrino et al., 1992; Gallese et al., 1996; Iacoboni, 2009). Mirror neuron activation was also witnessed in macaque monkeys when they both performed a paper tearing hand action, as well as visualized and heard this hand action delivered (e.g., paper ripping). Control sounds, such as unrelated noise and monkey vocalizations, did not elicit VPA neuron firing (Kohler et al., 2002). However, mirror neurons are not only concerned with hand-controlled actions. In fact, Ferrari et al. (2003) have corroborated that mirror neurons also have jurisdiction over facial actions (e.g., biting, sucking). It is possible that this system may play a role in contagious itch, just as it is a possible neural mechanism in contagious yawning (Ikoma et al., 2006; Miller et al., 2012; Haker et al., 2013; Gallup and Eldakar, 2013). It still remains unclear if mirror neurons are actually activated during contagious itching. Therefore, this hypothesis should be investigated in future studies. Moreover, it would be interesting to explore whether it is an action-based mirror system or rather a feeling-based mirror system that is crucial for contagious itch. Regarding this question, Holle et al. (2012) argue that a feeling-based system has the more crucial role in contagious itch, due to the fact that the insula, a region associated with the affective components of bodily sensations, showed the more sustained activity during contagious itch. Supporting this idea, Papoiu et al. (2011) and Ward et al. (2013) have shown observers scratched body areas that differed in body locations than those they observed. Lloyd et al. (2013) demonstrated that viewing images of insects was able to induce more intense itch than viewing scratching behavior, which again emphasizes that it is not the action that is important to experience itch due to visual itch cues, but rather the negative affect that is evoked when seeing itch stimuli.

Another possible reason for contagious itch is classical conditioning. This is especially conceivable when even the simple visual presentation of itch inducing objects (e.g., ants, mosquito bites) become triggering factors of itch. According to the principle of classical conditioning (Pavlov, 2003), the pairing of an unconditioned stimulus (UCS; in this case e.g., the histamine release following a mosquito bite) with an originally neutral stimulus (NS; seeing the mosquito on the skin) can lead to a provocation of itch just from seeing the mosquito on the skin alone. In this scenario, the image of the mosquito has become a conditioned stimulus (CS). It would be interesting to test the hypothesis that contagious itch occurs due to classical conditioning processes in a study in which a pruritogen (e.g., histamine) is paired with a NS. After repeating the simultaneous presentation of these stimuli a few times, the originally NS should also be able to evoke itch and a scratch response by itself. A similar study design was used by Russell et al. (1984), who could show that histamine release can be “learned”. Here, guinea pigs were first sensitized to bovine serum albumin (BSA), which acted as the UCS of histamine release. Afterwards, BSA together with a certain odor (fishy or sulfur) was injected in all guinea pigs. Moreover, as a control condition, saline was injected in combination with a different odor. The odor paired with histamine was counterbalanced between animals. After a few weeks, the plasma histamine concentration in response to the odor that was combined with histamine beforehand was higher than the histamine release due to the odor, which was given in the control condition. Similarly, Jordan and Whitlock (1972, 1974) were able to show that a scratch response could be conditioned in AD-patients and healthy controls. In these studies, a tone was used as the UCS, which was presented together with an itch stimulus (electrodes). Interestingly, in these studies, chronic itch patients reacted with a higher conditioned scratch response than healthy controls. This result is in line with the findings of two former studies, in which Papoiu et al. (2011) and Schut et al. (2014) found that visual itch cues, which in our opinion act as CS, also led to a higher scratch response in patients with chronic itch than in healthy controls.

Combining the approaches of classical conditioning and the activation of mirror neurons, Heyes (2010) proposes that mirror neurons are not hereditary, but rather a “byproduct” of associative learning. In the case of contagious itch, one could argue that because the pairing of itch and audiovisual cues (e.g., seeing a hand scratching or affected skin) has occurred much more frequently in chronic itch patients than in healthy controls, the mirror neurons firing during contagious itch should also be more active in chronic itch patients than in healthy controls. It would certainly be interesting to compare the activity of mirror neurons in patients with itchy skin diseases with their activity in healthy controls during contagious itch assuming that the presentation of visual itch stimuli would lead to higher mirror neuron activation in chronic itch patients than in healthy controls.

## Mood and personality as moderators of audio-visually transmitted itch

Whether one is susceptible to audiovisual itch stimuli also depends on personality characteristics and on the person’s mood. Ogden and Zoukas (2009) found a significant positive correlation between itch and anxiety. The more the students felt itchy after watching an itch inducing video, the more they also reported to experience anxiety. Of course, this relationship does not conclude whether anxiety precedes or follows itch. It only suggests that there is a correlation between psychological states and clinical symptoms, which is an interesting finding in itself.

Neuroticism (defined as emotional instability) is a personality trait that has been shown to be associated with the itch intensity induced by visual stimuli in healthy participants (Holle et al., 2012). Persons, who are neurotic, are more prone to experience negative emotions like fear, anger, disgust and embarrassment (McCrae and Costa, 2010). Holle et al. (2012) found a significant positive relationship between this trait and the itch increase due to watching another person scratching. Though this relationship was only investigated in healthy participants, but not in patients suffering from chronic itch.

The relationship between visually transmitted itch and psychological variables was further strengthened by a study in which depression and other personality characteristics were shown to be significant predictors of the extent of self-reported induced itch in AD-patients (Schut et al., 2014). AD-patients who reported personality traits of not being cooperative (low agreeableness) and at the same time high scores on the scale “public self-consciousness”, showed a higher number of scratch movements than patients who did not show this psychological phenotype (Schut et al., 2014). Interestingly, this relationship could only be shown in AD-patients, but not in healthy controls (Schut et al., 2014).

Although these findings suggest a relationship between negative mood, personality characteristics and an increase in itch/scratching, we cannot assume that persons with a certain personality will definitely develop an itchy skin disease. It is important to point out that there are many factors contributing to the development and maintenance of a disease. A specific combination of personality characteristics and moods should only be seen as one factor that can aggravate the experience of itch in an experimental setting.

## Next steps in the research on visually transmitted itch

There are still many open questions in the research of contagious itch. Although there are initial hypotheses as to why people feel an itch sensation when they observe the scratching behavior of others, these theories need to be further tested and verified. One explanation of contagious itch was presented by Niemeier et al. (2000), who postulated that itch-inducing stimuli may stimulate histamine release (Niemeier et al., 2000). Yet, it can also be assumed that contagious itch is actually a brain-phenomenon leading “only” to brain activation of certain interoceptive brain areas (e.g., the insula), and that no pruritogens are released.

Additional, functional brain imaging studies investigating brain substrates involved in contagious itch in healthy and chronic itch patients during audiovisually induced itch are rare (Mochizuki et al., 2014a) and therefore required. Mochizuki et al. (2013) displayed pictures of itch-related situations (e.g., mosquito bite; skin diseases) to subjects who underwent FMRI scans and were asked to simultaneously internally focus on their own analogous body parts. The pictures led to significantly higher brain activity in the left prefrontal cortex, left fusiform gyrus, bilateral anterior insular cortex, left orbitofrontal cortex, left supplementary motor area, left striatum, bilateral thalamus and bilateral cerebellum, as well as to significantly greater feelings of itch compared to control pictures showing unaffected skin. Holle et al. (2012) could show that the thalamus, primary somatosensory cortex, premotor cortex and insula were activated while watching videos of other people scratching. Even though these two studies give preliminary insight into which brain areas are activated when experiencing contagious itch, they only included healthy controls and it is unclear whether the same brain areas would be activated in chronic itch patients. It is reasonable to assume that contagious itch evokes a greater negative emotional response in chronic itch patients than in healthy controls, which might lead to a greater activation of the limbic system. Moreover, we would assume the insular and anterior cingulate cortices to be more activated in patients with chronic itch, because activation in these brain areas was associated with empathy for other bodily sensations like pain and touch (Bufalari and Ionta, 2013). Empathy for the other person scratching should be more pronounced in patients suffering from chronic itch compared to healthy controls. This hypothesis should be investigated in future studies.

Other important areas to investigate are whether demographic variables like age and gender have an effect on this behavioral phenomenon. Because neuroticism is more pronounced in younger than in older adults (McCrae et al., 1999) and is associated with contagious itch (Holle et al., 2012), it would also be reasonable to assume that younger subjects are more susceptible to contagious itch. Also, we hypothesize that due to higher empathy (e.g., Wilson et al., 2012), women may be more susceptible to visual itch cues. It has already been shown that women report higher itch intensities than men on visual analog scales (Ständer et al., 2013).

Another meaningful next step in the field of contagious itch is to investigate the effectiveness of strategies or treatments on reducing itch intensity due to visual itch cues. One possible method to diminish contagious itch is to prevent patients with itchy skin diseases from sharing a room with each other in the hospital. Another idea would be to extinct the classically conditioned response of scratching by pairing the visual itch cue with an itch-relieving cue. For instance, an itch-inducing image could be paired with that of a soothing skin ointment. Another psychological approach might be to lower the induced scratch response by teaching habit reversal techniques (Azrin and Nunn, 1973; Rosenbaum and Ayllon, 1981; Melin et al., 1986; Norén and Melin, 1989), which patients could then perform when being exposed to itch stimuli or to reduce stress during exposure to visual itch cues by e.g., the practice of relaxation techniques like progressive muscle relaxation (Jacobson, 2011).

## Conclusion

Contagious itch is a common behavioral phenomenon that evokes unique brain activations. More research is required to understand the neural substrates involved in contagious itch.

## Conflict of interest statement

The authors declare that the research was conducted in the absence of any commercial or financial relationships that could be construed as a potential conflict of interest.
